# Feasibility of Actigraphy in a Longitudinal Study of Patients with Bipolar Disorder

**DOI:** 10.1192/j.eurpsy.2025.1088

**Published:** 2025-08-26

**Authors:** C. Sommerhoff, M. Bort, G. Fico, T. Fernández-Plaza, Y. Rivas, V. Ruiz, M. De Prisco, V. Oliva, E. Vieta, A. Murru

**Affiliations:** 1 Department of Medicine, Faculty of Medicine and Health Sciences, University of Barcelona; 2 Bipolar and Depressive Disorders Unit, Hospital Clinic de Barcelona; 3 Institut d’Investigacions Biomèdiques August Pi i Sunyer (IDIBAPS), Barcelona; 4 Centro de Investigación Biomédica en Red de Salud Mental (CIBERSAM), Instituto de Salud Carlos III, Madrid; 5 Department of Medicine, Faculty of Medicine and Health Sciences; 6 Mental Health Outpatients Unit, Hospital Clinic de Barcelona, Barcelona, Spain

## Abstract

**Introduction:**

Bipolar disorder (BD) is a severe psychiatric disorder characterized by recurring acute mood episodes of depression or euphoria, alternating with phases of euthymia, with a prevalence in the general population of 2%. Individuals with BD frequently experience disruptions in sleep and circadian rhythms, present during both euthymic and acute mood phases, yet these disturbances are challenging to measure objectively. Tracking these changes can offer insights into the progression of BD and potential risk for mood relapse. Actigraphy, a non-invasive monitoring technique, holds promise for capturing objective, real-world data on sleep and circadian patterns, thereby aiding in the assessment and management of BD.

**Objectives:**

This study aims to assess the feasibility of using actigraphy as a tool for monitoring sleep-wake patterns and physical activity levels in patients with BD over a longitudinal period.

**Methods:**

Within The Bipolar Exposome-Gene Interaction Naturalistic (BEGIN) project we conducted a longitudinal study of individuals diagnosed with BD using wrist-worn actigraphy (Withings Steel HR) to collect data on daily activity and sleep parameters over 12 months. Feasibility was evaluated based on participant adherence to the actigraphy protocol, data quality, technical challenges, and acceptability by patients and clinicians.

**Results:**

Results indicated satisfactory enrolment rate (N=87, 66.4%) in the study and compliance with the use of actigraphy (N= 59, 67.8%). The main reasons for not accepting the study were non-response to contacting efforts (43.2%) and lack of interest (24.3%). No age differences were found between individuals who accepted the study and those who did not. A high retention rate was obtained (N=80, 92%) with the main reason for drop-off being the perception of intense follow-up within the study. Regarding actigraphy, younger individuals were more likely to accept its use (t = -2.066, p-value = 0.043) and the main reason for non-adherence was rash development (42.9%). Patient feedback highlighted the ease of use and minimal disruption of daily life.

**Conclusions:**

In conclusion, actigraphy is a feasible and effective tool for continuous monitoring in BD, offering potential for improving the understanding of mood episodes and treatment efficacy in future studies. Further exploration of actigraphy in larger cohorts and its integration with other physiological measures is recommended.

**Disclosure of Interest:**

None Declared

